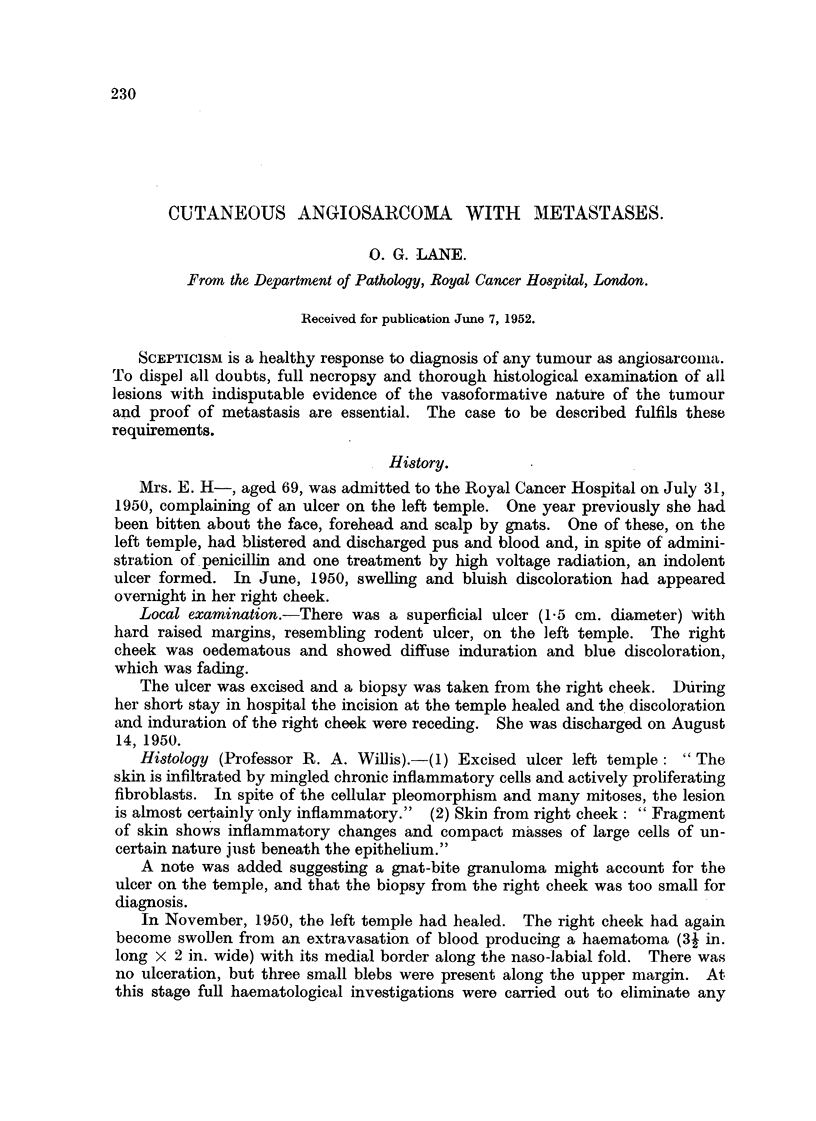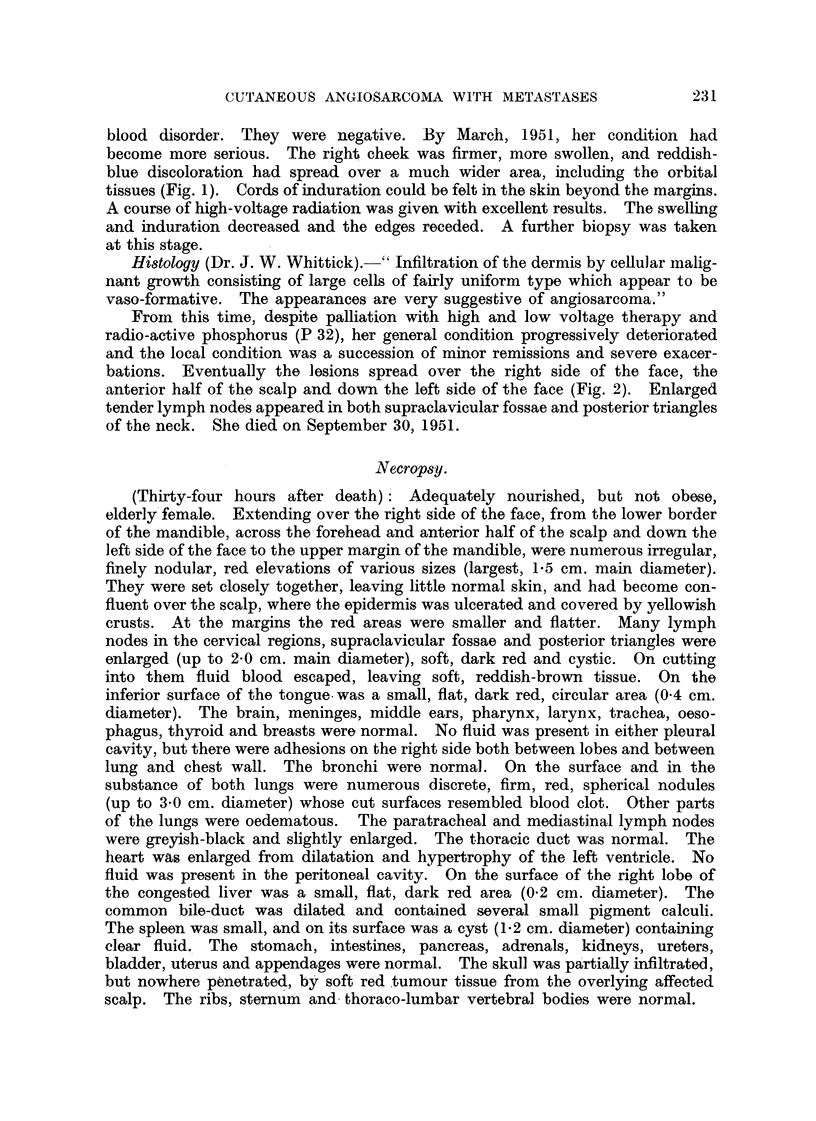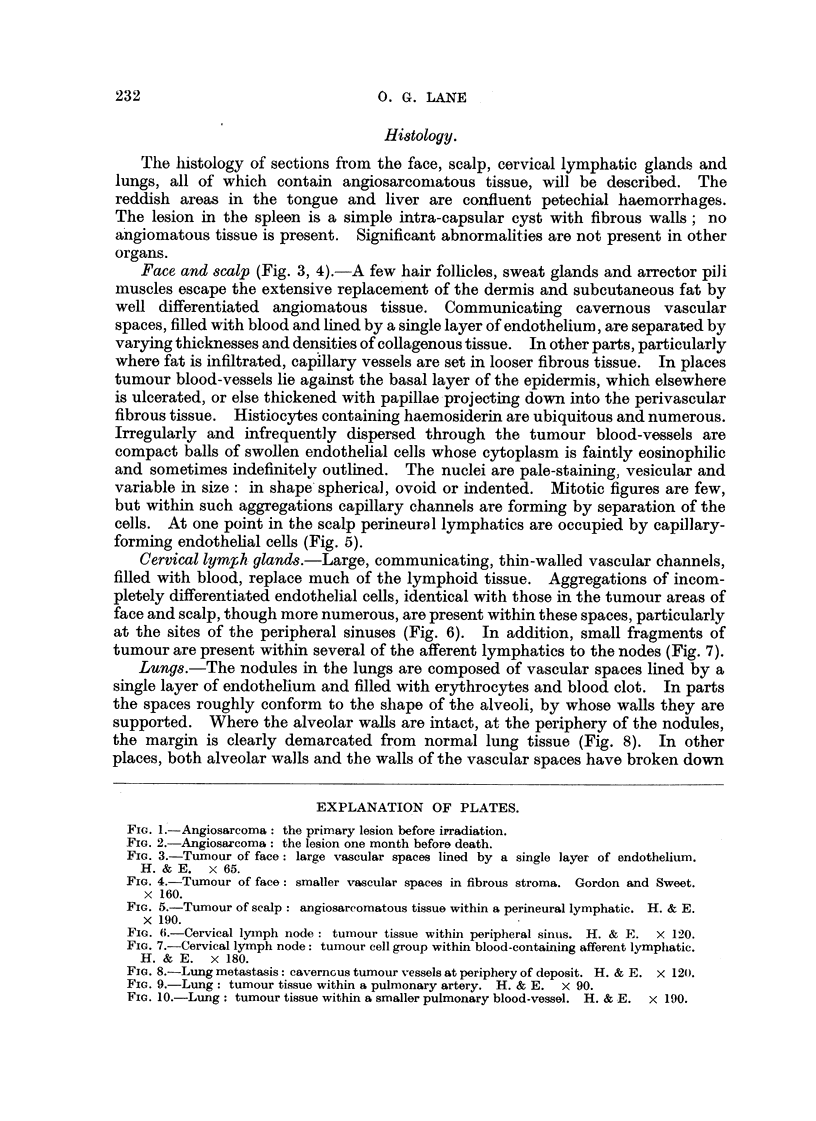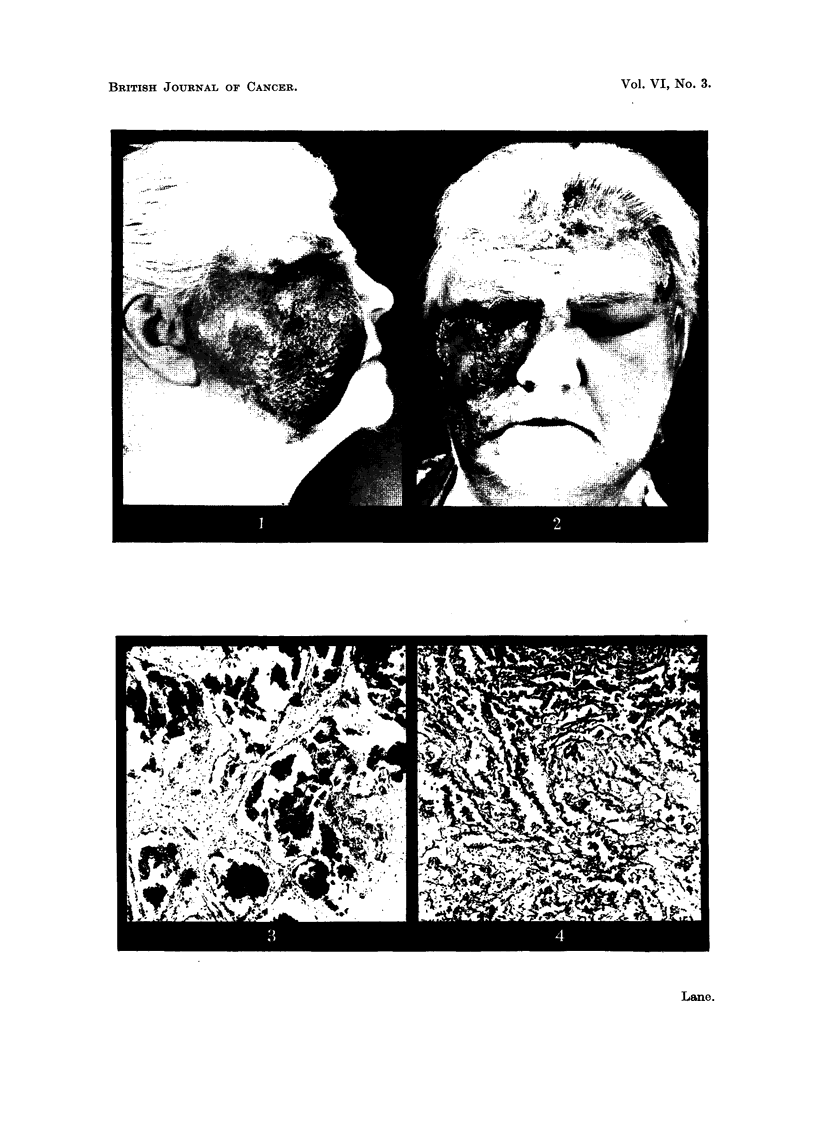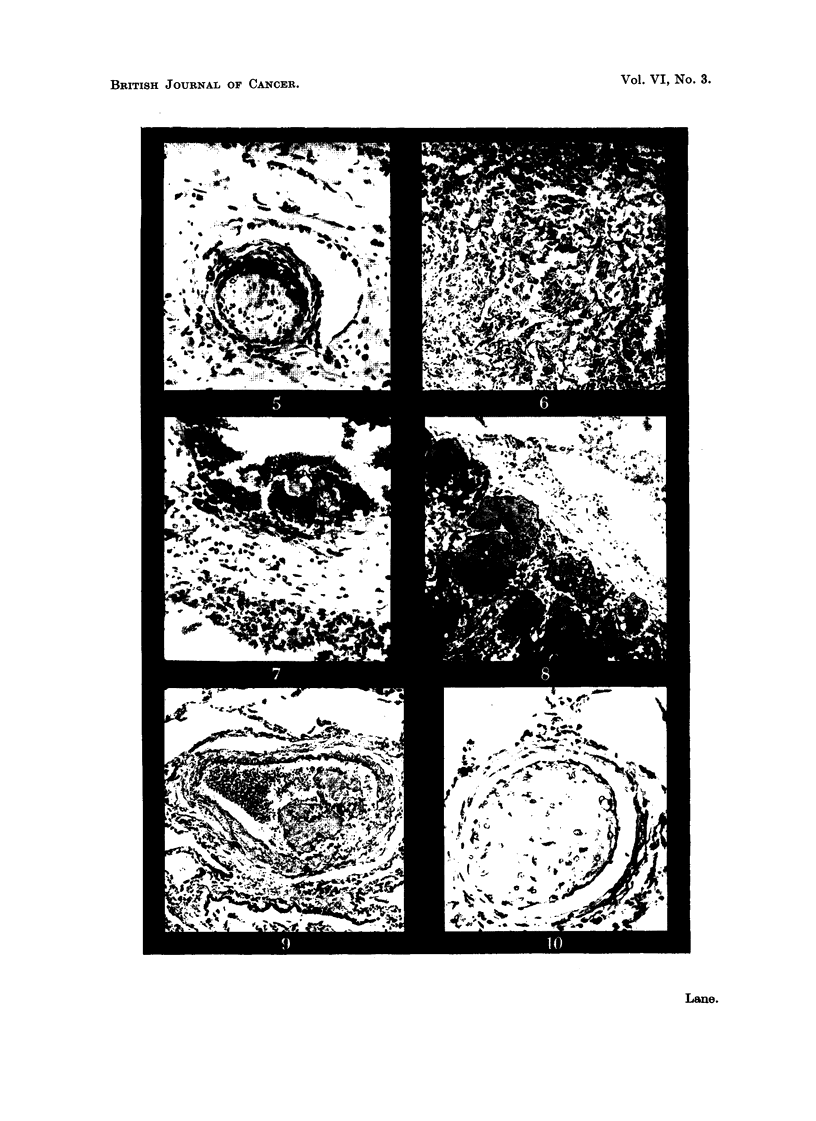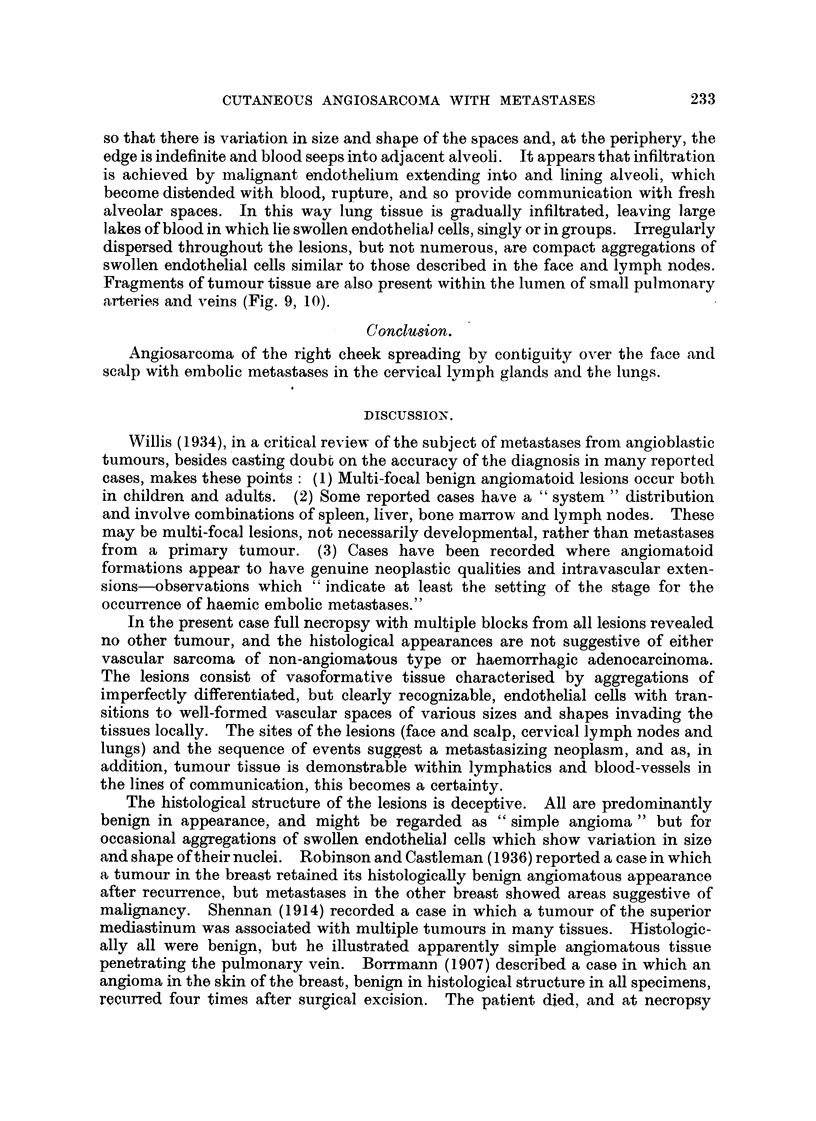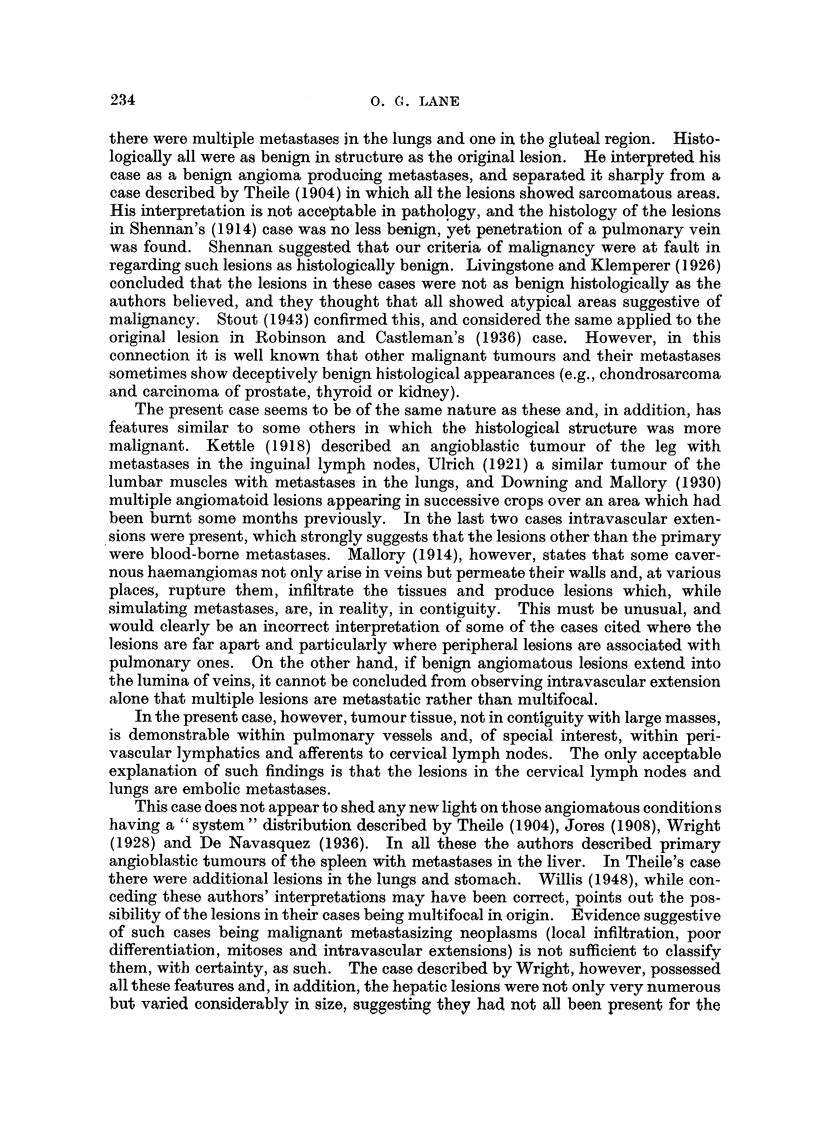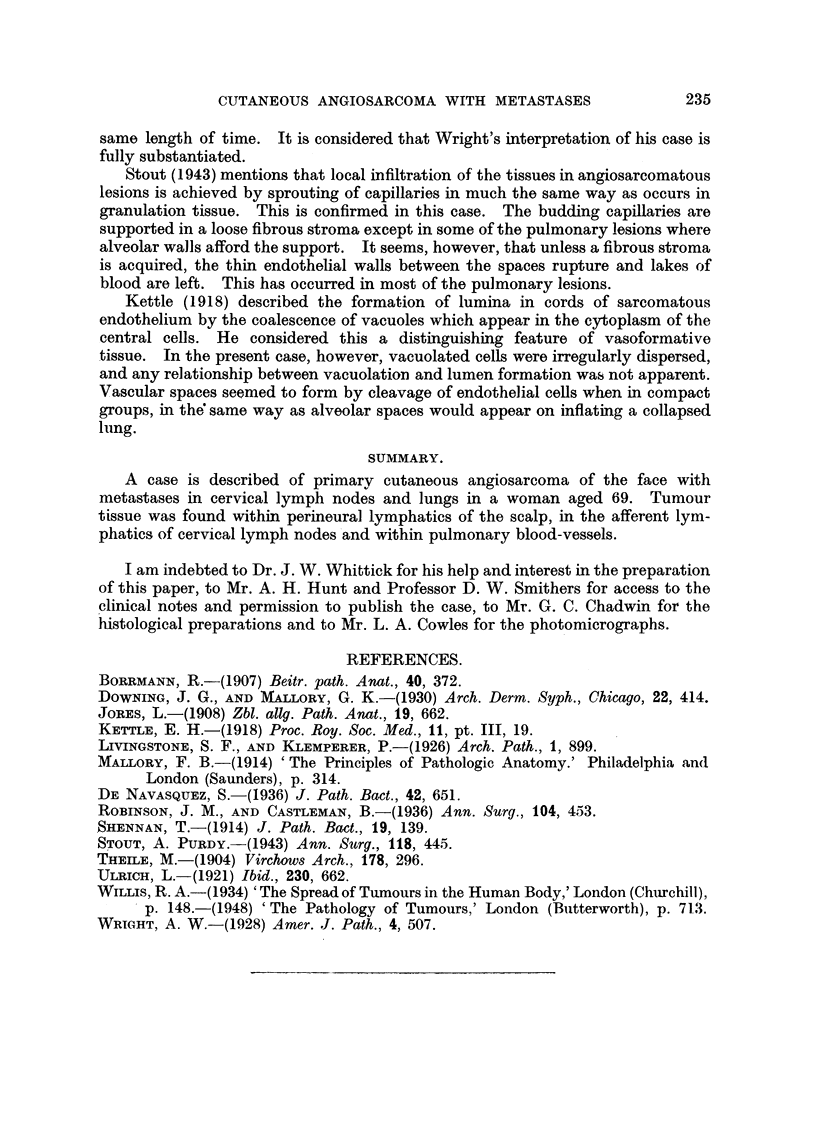# Cutaneous Angiosarcoma with Metastases

**DOI:** 10.1038/bjc.1952.25

**Published:** 1952-09

**Authors:** O. G. Lane

## Abstract

**Images:**


### CUTANEOUS ANGIOSARCOMA WITH METASTASES.

O. G. LANE.

*From the Department of Pathology, Royal Cancer Hospital, London.*

Received for publication June 7, 1952.

SCEPTICISM is a healthy response to diagnosis of any tumour as angiosarcoma. To dispel all doubts, full necropsy and thorough histological examination of all lesions with indisputable evidence of the vasoformative nature of the tumour and proof of metastasis are essential. The case to be described fulfils these requirements.

### *History.*

Mrs. E. H—, aged 69, was admitted to the Royal Cancer Hospital on July 31, 1950, complaining of an ulcer on the left temple. One year previously she had been bitten about the face, forehead and scalp by gnats. One of these, on the left temple, had blistered and discharged pus and blood and, in spite of administration of penicillin and one treatment by high voltage radiation, an indolent ulcer formed. In June, 1950, swelling and bluish discoloration had appeared overnight in her right cheek.

*Local examination.*—There was a superficial ulcer (1·5 cm. diameter) with hard raised margins, resembling rodent ulcer, on the left temple. The right cheek was oedematous and showed diffuse induration and blue discoloration, which was fading.

The ulcer was excised and a biopsy was taken from the right cheek. During her short stay in hospital the incision at the temple healed and the discoloration and induration of the right cheek were receding. She was discharged on August 14, 1950.

*Histology* (Professor R. A. Willis).—(1) Excised ulcer left temple: "The skin is infiltrated by mingled chronic inflammatory cells and actively proliferating fibroblasts. In spite of the cellular pleomorphism and many mitoses, the lesion is almost certainly only inflammatory." (2) Skin from right cheek: "Fragment of skin shows inflammatory changes and compact masses of large cells of uncertain nature just beneath the epithelium."

A note was added suggesting a gnat-bite granuloma might account for the ulcer on the temple, and that the biopsy from the right cheek was too small for diagnosis.

In November, 1950, the left temple had healed. The right cheek had again become swollen from an extravasation of blood producing a haematoma ($3\frac{1}{2}$ in. long $\times$ 2 in. wide) with its medial border along the naso-labial fold. There was no ulceration, but three small blebs were present along the upper margin. At this stage full haematological investigations were carried out to eliminate any blood disorder. They were negative. By March, 1951, her condition had become more serious. The right cheek was firmer, more swollen, and reddish-blue discoloration had spread over a much wider area, including the orbital tissues (Fig. 1). Cords of induration could be felt in the skin beyond the margins. A course of high-voltage radiation was given with excellent results. The swelling and induration decreased and the edges receded. A further biopsy was taken at this stage.

*Histology* (Dr. J. W. Whittick).—" Infiltration of the dermis by cellular malignant growth consisting of large cells of fairly uniform type which appear to be vaso-formative. The appearances are very suggestive of angiosarcoma."

From this time, despite palliation with high and low voltage therapy and radio-active phosphorus (P 32), her general condition progressively deteriorated and the local condition was a succession of minor remissions and severe exacerbations. Eventually the lesions spread over the right side of the face, the anterior half of the scalp and down the left side of the face (Fig. 2). Enlarged tender lymph nodes appeared in both supraclavicular fossae and posterior triangles of the neck. She died on September 30, 1951.

### *Necropsy.*

(Thirty-four hours after death): Adequately nourished, but not obese, elderly female. Extending over the right side of the face, from the lower border of the mandible, across the forehead and anterior half of the scalp and down the left side of the face to the upper margin of the mandible, were numerous irregular, finely nodular, red elevations of various sizes (largest, 1·5 cm. main diameter). They were set closely together, leaving little normal skin, and had become confluent over the scalp, where the epidermis was ulcerated and covered by yellowish crusts. At the margins the red areas were smaller and flatter. Many lymph nodes in the cervical regions, supraclavicular fossae and posterior triangles were enlarged (up to 2·0 cm. main diameter), soft, dark red and cystic. On cutting into them fluid blood escaped, leaving soft, reddish-brown tissue. On the inferior surface of the tongue was a small, flat, dark red, circular area (0·4 cm. diameter). The brain, meninges, middle ears, pharynx, larynx, trachea, oesophagus, thyroid and breasts were normal. No fluid was present in either pleural cavity, but there were adhesions on the right side both between lobes and between lung and chest wall. The bronchi were normal. On the surface and in the substance of both lungs were numerous discrete, firm, red, spherical nodules (up to 3·0 cm. diameter) whose cut surfaces resembled blood clot. Other parts of the lungs were oedematous. The paratracheal and mediastinal lymph nodes were greyish-black and slightly enlarged. The thoracic duct was normal. The heart was enlarged from dilatation and hypertrophy of the left ventricle. No fluid was present in the peritoneal cavity. On the surface of the right lobe of the congested liver was a small, flat, dark red area (0·2 cm. diameter). The common bile-duct was dilated and contained several small pigment calculi. The spleen was small, and on its surface was a cyst (1·2 cm. diameter) containing clear fluid. The stomach, intestines, pancreas, adrenals, kidneys, ureters, bladder, uterus and appendages were normal. The skull was partially infiltrated, but nowhere penetrated, by soft red tumour tissue from the overlying affected scalp. The ribs, sternum and thoraco-lumbar vertebral bodies were normal.

### *Histology.*

The histology of sections from the face, scalp, cervical lymphatic glands and lungs, all of which contain angiosarcomatous tissue, will be described. The reddish areas in the tongue and liver are confluent petechial haemorrhages. The lesion in the spleen is a simple intra-capsular cyst with fibrous walls ; no angiomatous tissue is present. Significant abnormalities are not present in other organs.

*Face and scalp* (Fig. 3, 4).—A few hair follicles, sweat glands and arrector pili muscles escape the extensive replacement of the dermis and subcutaneous fat by well differentiated angiomatous tissue. Communicating cavernous vascular spaces, filled with blood and lined by a single layer of endothelium, are separated by varying thicknesses and densities of collagenous tissue. In other parts, particularly where fat is infiltrated, capillary vessels are set in looser fibrous tissue. In places tumour blood-vessels lie against the basal layer of the epidermis, which elsewhere is ulcerated, or else thickened with papillae projecting down into the perivascular fibrous tissue. Histiocytes containing haemosiderin are ubiquitous and numerous. Irregularly and infrequently dispersed through the tumour blood-vessels are compact balls of swollen endothelial cells whose cytoplasm is faintly eosinophilic and sometimes indefinitely outlined. The nuclei are pale-staining, vesicular and variable in size : in shape spherical, ovoid or indented. Mitotic figures are few, but within such aggregations capillary channels are forming by separation of the cells. At one point in the scalp perineural lymphatics are occupied by capillary-forming endothelial cells (Fig. 5).

*Cervical lymph glands.*—Large, communicating, thin-walled vascular channels, filled with blood, replace much of the lymphoid tissue. Aggregations of incompletely differentiated endothelial cells, identical with those in the tumour areas of face and scalp, though more numerous, are present within these spaces, particularly at the sites of the peripheral sinuses (Fig. 6). In addition, small fragments of tumour are present within several of the afferent lymphatics to the nodes (Fig. 7).

*Lungs.*—The nodules in the lungs are composed of vascular spaces lined by a single layer of endothelium and filled with erythrocytes and blood clot. In parts the spaces roughly conform to the shape of the alveoli, by whose walls they are supported. Where the alveolar walls are intact, at the periphery of the nodules, the margin is clearly demarcated from normal lung tissue (Fig. 8). In other places, both alveolar walls and the walls of the vascular spaces have broken down

---

EXPLANATION OF PLATES.

FIG. 1.—Angiosarcoma : the primary lesion before irradiation.
FIG. 2.—Angiosarcoma : the lesion one month before death.
FIG. 3.—Tumour of face : large vascular spaces lined by a single layer of endothelium. H. & E. × 65.
FIG. 4.—Tumour of face : smaller vascular spaces in fibrous stroma. Gordon and Sweet. × 160.
FIG. 5.—Tumour of scalp : angiosarcomatous tissue within a perineural lymphatic. H. & E. × 190.
FIG. 6.—Cervical lymph node : tumour tissue within peripheral sinus. H. & E. × 120.
FIG. 7.—Cervical lymph node : tumour cell group within blood-containing afferent lymphatic. H. & E. × 180.
FIG. 8.—Lung metastasis : cavernous tumour vessels at periphery of deposit. H. & E. × 120.
FIG. 9.—Lung : tumour tissue within a pulmonary artery. H. & E. × 90.
FIG. 10.—Lung : tumour tissue within a smaller pulmonary blood-vessel. H. & E. × 190.

1

2

3

4

Lane.

5

6

7

8

9

10

Lane.

so that there is variation in size and shape of the spaces and, at the periphery, the edge is indefinite and blood seeps into adjacent alveoli. It appears that infiltration is achieved by malignant endothelium extending into and lining alveoli, which become distended with blood, rupture, and so provide communication with fresh alveolar spaces. In this way lung tissue is gradually infiltrated, leaving large lakes of blood in which lie swollen endothelial cells, singly or in groups. Irregularly dispersed throughout the lesions, but not numerous, are compact aggregations of swollen endothelial cells similar to those described in the face and lymph nodes. Fragments of tumour tissue are also present within the lumen of small pulmonary arteries and veins (Fig. 9, 10).

*Conclusion.*

Angiosarcoma of the right cheek spreading by contiguity over the face and scalp with embolic metastases in the cervical lymph glands and the lungs.

### DISCUSSION.

Willis (1934), in a critical review of the subject of metastases from angioblastic tumours, besides casting doubt on the accuracy of the diagnosis in many reported cases, makes these points : (1) Multi-focal benign angiomatoid lesions occur both in children and adults. (2) Some reported cases have a " system " distribution and involve combinations of spleen, liver, bone marrow and lymph nodes. These may be multi-focal lesions, not necessarily developmental, rather than metastases from a primary tumour. (3) Cases have been recorded where angiomatoid formations appear to have genuine neoplastic qualities and intravascular extensions—observations which " indicate at least the setting of the stage for the occurrence of haemic embolic metastases."

In the present case full necropsy with multiple blocks from all lesions revealed no other tumour, and the histological appearances are not suggestive of either vascular sarcoma of non-angiomatous type or haemorrhagic adenocarcinoma. The lesions consist of vasoformative tissue characterised by aggregations of imperfectly differentiated, but clearly recognizable, endothelial cells with transitions to well-formed vascular spaces of various sizes and shapes invading the tissues locally. The sites of the lesions (face and scalp, cervical lymph nodes and lungs) and the sequence of events suggest a metastasizing neoplasm, and as, in addition, tumour tissue is demonstrable within lymphatics and blood-vessels in the lines of communication, this becomes a certainty.

The histological structure of the lesions is deceptive. All are predominantly benign in appearance, and might be regarded as " simple angioma " but for occasional aggregations of swollen endothelial cells which show variation in size and shape of their nuclei. Robinson and Castleman (1936) reported a case in which a tumour in the breast retained its histologically benign angiomatous appearance after recurrence, but metastases in the other breast showed areas suggestive of malignancy. Shennan (1914) recorded a case in which a tumour of the superior mediastinum was associated with multiple tumours in many tissues. Histologically all were benign, but he illustrated apparently simple angiomatous tissue penetrating the pulmonary vein. Borrmann (1907) described a case in which an angioma in the skin of the breast, benign in histological structure in all specimens, recurred four times after surgical excision. The patient died, and at necropsy there were multiple metastases in the lungs and one in the gluteal region. Histologically all were as benign in structure as the original lesion. He interpreted his case as a benign angioma producing metastases, and separated it sharply from a case described by Theile (1904) in which all the lesions showed sarcomatous areas. His interpretation is not acceptable in pathology, and the histology of the lesions in Shennan's (1914) case was no less benign, yet penetration of a pulmonary vein was found. Shennan suggested that our criteria of malignancy were at fault in regarding such lesions as histologically benign. Livingstone and Klemperer (1926) concluded that the lesions in these cases were not as benign histologically as the authors believed, and they thought that all showed atypical areas suggestive of malignancy. Stout (1943) confirmed this, and considered the same applied to the original lesion in Robinson and Castleman's (1936) case. However, in this connection it is well known that other malignant tumours and their metastases sometimes show deceptively benign histological appearances (e.g., chondrosarcoma and carcinoma of prostate, thyroid or kidney).

The present case seems to be of the same nature as these and, in addition, has features similar to some others in which the histological structure was more malignant. Kettle (1918) described an angioblastic tumour of the leg with metastases in the inguinal lymph nodes, Ulrich (1921) a similar tumour of the lumbar muscles with metastases in the lungs, and Downing and Mallory (1930) multiple angiomatoid lesions appearing in successive crops over an area which had been burnt some months previously. In the last two cases intravascular extensions were present, which strongly suggests that the lesions other than the primary were blood-borne metastases. Mallory (1914), however, states that some cavernous haemangiomas not only arise in veins but permeate their walls and, at various places, rupture them, infiltrate the tissues and produce lesions which, while simulating metastases, are, in reality, in contiguity. This must be unusual, and would clearly be an incorrect interpretation of some of the cases cited where the lesions are far apart and particularly where peripheral lesions are associated with pulmonary ones. On the other hand, if benign angiomatous lesions extend into the lumina of veins, it cannot be concluded from observing intravascular extension alone that multiple lesions are metastatic rather than multifocal.

In the present case, however, tumour tissue, not in contiguity with large masses, is demonstrable within pulmonary vessels and, of special interest, within perivascular lymphatics and afferents to cervical lymph nodes. The only acceptable explanation of such findings is that the lesions in the cervical lymph nodes and lungs are embolic metastases.

This case does not appear to shed any new light on those angiomatous conditions having a "system" distribution described by Theile (1904), Jores (1908), Wright (1928) and De Navasquez (1936). In all these the authors described primary angioblastic tumours of the spleen with metastases in the liver. In Theile's case there were additional lesions in the lungs and stomach. Willis (1948), while conceding these authors' interpretations may have been correct, points out the possibility of the lesions in their cases being multifocal in origin. Evidence suggestive of such cases being malignant metastasizing neoplasms (local infiltration, poor differentiation, mitoses and intravascular extensions) is not sufficient to classify them, with certainty, as such. The case described by Wright, however, possessed all these features and, in addition, the hepatic lesions were not only very numerous but varied considerably in size, suggesting they had not all been present for the same length of time. It is considered that Wright's interpretation of his case is fully substantiated.

Stout (1943) mentions that local infiltration of the tissues in angiosarcomatous lesions is achieved by sprouting of capillaries in much the same way as occurs in granulation tissue. This is confirmed in this case. The budding capillaries are supported in a loose fibrous stroma except in some of the pulmonary lesions where alveolar walls afford the support. It seems, however, that unless a fibrous stroma is acquired, the thin endothelial walls between the spaces rupture and lakes of blood are left. This has occurred in most of the pulmonary lesions.

Kettle (1918) described the formation of lumina in cords of sarcomatous endothelium by the coalescence of vacuoles which appear in the cytoplasm of the central cells. He considered this a distinguishing feature of vasoformative tissue. In the present case, however, vacuolated cells were irregularly dispersed, and any relationship between vacuolation and lumen formation was not apparent. Vascular spaces seemed to form by cleavage of endothelial cells when in compact groups, in the same way as alveolar spaces would appear on inflating a collapsed lung.

### SUMMARY.

A case is described of primary cutaneous angiosarcoma of the face with metastases in cervical lymph nodes and lungs in a woman aged 69. Tumour tissue was found within perineural lymphatics of the scalp, in the afferent lymphatics of cervical lymph nodes and within pulmonary blood-vessels.

I am indebted to Dr. J. W. Whittick for his help and interest in the preparation of this paper, to Mr. A. H. Hunt and Professor D. W. Smithers for access to the clinical notes and permission to publish the case, to Mr. G. C. Chadwin for the histological preparations and to Mr. L. A. Cowles for the photomicrographs.

### REFERENCES.

Borrmann, R.—(1907) *Beitr. path. Anat.*, **40**, 372.
Downing, J. G., and Mallory, G. K.—(1930) *Arch. Derm. Syph., Chicago,* **22**, 414.
Jores, L.—(1908) *Zbl. allg. Path. Anat.*, **19**, 662.
Kettle, E. H.—(1918) *Proc. Roy. Soc. Med.*, **11**, pt. III, 19.
Livingstone, S. F., and Klemperer, P.—(1926) *Arch. Path.*, **1**, 899.
Mallory, F. B.—(1914) 'The Principles of Pathologic Anatomy.' Philadelphia and London (Saunders), p. 314.
De Navasquez, S.—(1936) *J. Path. Bact.*, **42**, 651.
Robinson, J. M., and Castleman, B.—(1936) *Ann. Surg.*, **104**, 453.
Shennan, T.—(1914) *J. Path. Bact.*, **19**, 139.
Stout, A. Purdy.—(1943) *Ann. Surg.*, **118**, 445.
Theile, M.—(1904) *Virchows Arch.*, **178**, 296.
Ulrich, L.—(1921) *Ibid.*, **230**, 662.
Willis, R. A.—(1934) 'The Spread of Tumours in the Human Body,' London (Churchill), p. 148.—(1948) 'The Pathology of Tumours,' London (Butterworth), p. 713.
Wright, A. W.—(1928) *Amer. J. Path.*, **4**, 507.